# Does the Biological Response to Fetal Hypoxia Involve Angiogenesis, Placental Enlargement and Preeclampsia?

**DOI:** 10.1111/ppe.12283

**Published:** 2016-04-01

**Authors:** Anne Eskild, Ellen Marie Strøm‐Roum, Camilla Haavaldsen

**Affiliations:** ^1^ Department of Obstetrics and GynecologyAkershus University HospitalLørenskogNorway; ^2^ Institute of Clinical MedicineUniversity of OsloOsloNorway

From an evolutionary point of view, an overriding biological priority is to ensure offspring survival. Oxygen is essential for the growing fetus, and during pregnancy the fetus has an increasing demand for oxygen. If the oxygen supply to the fetus is threatened, it is likely that compensatory mechanisms are activated.

Impaired development of the placenta in early pregnancy is assumed to be the main cause of feto‐placental hypoxia and preeclampsia. However, it is also plausible that medical conditions in the mother could lead to sub‐optimal supply to the feto‐placental unit. Feto‐placental hypoxia may in such mothers develop in the last part of pregnancy when the oxygen demand is highest. We propose that in pregnancies with sub‐optimal availability of oxygen in maternal blood, placental dysfunction may not be the primary cause of feto‐placental hypoxia and preeclampsia. On the contrary, in these pregnancies, increased placental growth may be important as a compensatory mechanism to improve oxygen uptake from the maternal circulation.

On the basis of results from population studies, we hypothesise that both impaired placental development in early pregnancy and insufficient oxygen supply from the maternal circulation later in pregnancy, may cause feto‐placental hypoxia and preeclampsia (Figure 1). In the following, we will present arguments for such hypothesis.

**Figure 1:**
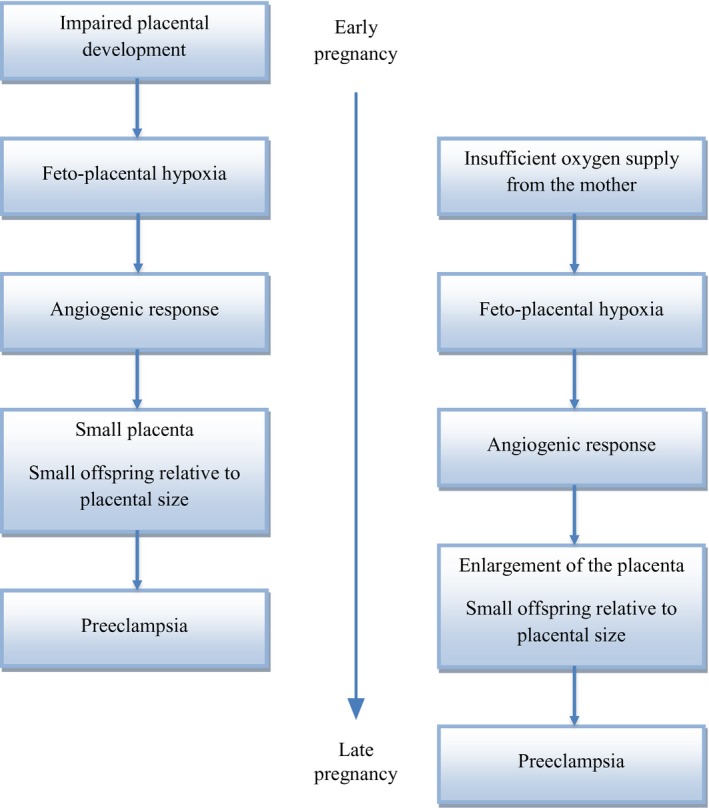
Proposed response to feto‐placental hypoxia caused by insufficient oxygen supply from the maternal circulation or by impaired placental development.

## Insufficient oxygen supply from the maternal circulation: a cause of fetal hypoxia?

Several conditions in the mother could be linked to sub‐optimal oxygen supply to the feto‐placental unit, and the biological mechanisms may vary. For instance, a mother with anaemia has low oxygen transport capacity, and the availability of oxygen in the intervillous space may be sub‐optimal. A mother with anaemia may therefore have reduced ability to provide her fetus with sufficient oxygen, in particular in the last part of pregnancy when the fetal demand is high. Pregnant women with heart or lung diseases and women who smoke are also likely to be sub‐optimal oxygen providers. Women with increased prevalence of atherosclerosis, such as women with diabetes may also have sub‐optimal ability to supply the feto‐placental unit with oxygen. Advanced age is associated with increased prevalence of cardiovascular disease and other medical conditions, and therefore older mothers may not be the best oxygen providers to their offspring. Some women may need much oxygen herself, such as women with high body mass index. In other pregnancies, the feto‐placental oxygen demand may be particularly high, such as in twin pregnancies. It is therefore conceivable that in some pregnancies, the oxygen supply from the maternal circulation may be sub‐optimal for the growing fetus.

## Enlargement of the placenta: a response to insufficient oxygen supply from the mother?

A large placenta is likely to have better capacity for oxygen uptake from the maternal circulation than a small placenta, since the number of chorionic villi may be higher, and thus the surface against the intervillous space may be larger.^1^, ^2^ In a normal placenta, growth and differentiation of villous trees take place throughout pregnancy. In the third trimester, numerous of terminal villi are produced by sprouting and growth of fetal capillaries within mature intermediate villi. It is likely that the branching of the placenta is driven by hypoxia.^3^ Hence, in placentas with sub‐optimal oxygen supply from the maternal circulation, the production of terminal villi and placental growth may be enhanced.

Enlarged placentas have been reported in pregnancies of women who may be poor oxygen providers, such as women with anaemia,^3^ diabetes,^4^ high body mass index^5^ or high age.^6^ Not only is there an absolute enlargement of the placenta in these pregnancies, but also the placenta is enlarged relative to the offspring size. The enlarged placenta in high risk pregnancies supports the hypothesis that placental growth could be a compensatory mechanism to improve the oxygen supply to the fetus.

Enlargement of the placenta may not ensure fetal well‐being in all pregnancies, but could be an indicator of fetal distress. Thus, a large placenta has been associated with low Apgar scores and increased admission rates to a neonatal intensive care unit for the infant.^7^ Placental enlargement has also been associated with increased risk of cardiovascular death in the adult life of the offspring.^8^

## Impaired early placental development as a cause of fetal hypoxia

If the development of the placenta is impaired in early pregnancy, the placental growth potential may be impaired. In such pregnancies, the placenta may remain relatively small with limited surface for oxygen exchange.^9^ Thus, a small placenta may have increasing difficulties with providing the offspring with oxygen as the pregnancy proceeds.

The causes of impaired placental development are insufficiently understood, but normal proliferation and differentiation of trophoblastic cells in early pregnancy may be driven by low‐oxygen concentration in the decidua.^10^ Premature differentiation of trophoblastic cells could be one explanation of impaired placental development, as it may limit villous sprouting of the placenta and thereby the potential for placental growth later in pregnancy.^1^ Thus, in pregnancies with impaired early placental development, growth, as a response to the increasing fetal oxygen demand during pregnancy, may not be possible. Pregnancies with a small placenta carry an increased risk for delivery of an infant small‐for‐gestational age and for stillbirth, particularly in the last part of pregnancy.^11^

## Large and small placentas: do they represent different underlying causes of fetal hypoxia?

We propose that both an enlarged placenta and a growth‐restricted placenta may be a sign of feto‐placental hypoxia. However, the underlying causes of the hypoxia may differ (Figure 1). When the placenta is large, the causes may be linked to insufficient oxygen supply from the maternal circulation. In these pregnancies, the placenta itself may be normal, and the excess growth could be a response to insufficient oxygen supply from the mother, as illustrated in pregnancies with maternal anaemia.^3^ When the placenta is small, the causes of the feto‐placental hypoxia may be linked to impaired placental development in early pregnancy.

Both in pregnancies with a small placenta,^11^ and in pregnancies with a large placenta,^4^, ^6^, ^12^ the placental weight relative to birthweight has been reported to be high (Figure 1). Thus, a high placental to birthweight ratio may be a sign of feto‐placental hypoxia, independent of the absolute size of the placenta or the fetus. In pregnancies with preeclampsia, the placental weight relative to birthweight seems to be high, independent of absolute placental size.^12^

## Increased angiogenesis and preeclampsia: responses to feto‐placental hypoxia of any cause?

Preeclampsia is assumed to be caused by placental hypoxia subsequent to impaired placental development in early pregnancy, and the maternal clinical characteristics of preeclampsia are hypertension and proteinuria. Population studies suggest, however, that both small and large placentas are overrepresented in pregnancies with preeclampsia, and in most pregnancies with preeclampsia, the placenta has a normal size.^13^ In term preeclampsia with a large offspring, the resistance in the uterine artery is lower than in preterm preeclampsia with a small offspring.^14^ These findings challenge the currently dominant hypothesis that impaired placental development in early pregnancy and subsequent placental dysfunction are the leading causes of hypoxia in preeclampsia.

One of the biological responses to hypoxia is development of new vessels, and this response is regulated by angiogenic factors.^15^ In pregnancy, many angiogenic factors are synthesised in the placenta, and hypoxia is likely to stimulate placental angiogenesis.^3^ However, placental responses to hypoxia may vary during pregnancy and are regulated in complex manners not yet understood.

Preeclampsia has been associated with altered maternal concentrations of angiogenic factors.^16^, ^17^ It is conceivable that the alterations of angiogenic factors seen in preeclampsia are responses to feto‐placental hypoxia of any cause. We propose that also insufficient oxygen supply from the maternal circulation may initiate an angiogenic response as seen in preeclampsia. The alteration in angiogenic factors^18^ and the increased risk of preeclampsia in twin pregnancies support such hypothesis. In twin pregnancies, the demand for oxygen from the maternal circulation is particularly high.

In pregnancies with preeclampsia to term or without fetal growth restriction, the alteration in angiogenic factors occurs later in pregnancy than in pregnancies with preterm preeclampsia.^16^, ^17^, ^19^ It is conceivable that angiogenic responses in pregnancies with a normal placenta could result in development of new chorionic villi and thereby placental enlargement. Development of new chorionic villi and thereby increased oxygen uptake,^2^ could possibly prevent fetal growth restriction and preeclampsia. In most pregnancies with placental enlargement preeclampsia does not occur, or preeclampsia occurs late in pregnancy.^6^, ^12^

In pregnancies with preeclampsia and a small‐for‐gestational age infant, the placenta is often small.^13^ In these pregnancies, the alterations in angiogenic factors are more prominent and occur earlier as compared to pregnancies, where preeclampsia occurs at term and the infant has normal size.^16^, ^17^ These findings could suggest that in pregnancies with hypoxia subsequent to impaired placental development, the angiogenic responses may not succeed in supporting normal placental or fetal growth.

In pregnancies with a small‐for‐gestational age infant without preeclampsia, the alterations in angiogenic factors are prominent and similar to the alterations found in preeclampsia.^20^ Thus, alteration in angiogenic factors may not be related to preeclampsia only, but be adequate responses to feto‐placental hypoxia. Human chorionic gonadotropin (hCG) concentrations may play an important role in stimulating proliferation of trophoblasts and angiogenesis in pregnancy,^21^ and hCG seems to interact with other angiogenic factors.^22^ In pregnancies with preeclampsia, the hCG levels increase in a similar pattern as the angiogenic factors soluble fms‐like tyrosine kinase‐1(sFlt‐1) and endoglin, suggesting that that these factors may be parts of the same angiogenic response to hypoxia.^17^

The increased blood pressure and endothelial permeability that is present in preeclampsia could theoretically increase the availability of oxygen to the placenta and thereby to the fetus. Increased diastolic blood pressure is associated with increased resistance in the maternal peripheral circulation, increased blood flow in the uterine artery and thus possibly increased blood flow to the intervillous space. The increased endothelial permeability in preeclampsia may increase the concentration of red blood cells in the maternal circulation.^23^ Thus, the haemoglobin concentrations in the intervillous space may increase and thereby the availability of oxygen for the feto‐placental unit may increase.^2^ Increased blood pressure and increased endothelial permeability may therefore represent adequate compensatory responses to feto‐placental hypoxia.

Women who develop preeclampsia have an increased prevalence of risk factors for hypertension prior to pregnancy, and women with preeclampsia have increased risk of hypertension after pregnancy.^24^ It is possible that similar angiogenic responses, as in preeclampsia, are linked to the development of hypertension also in individuals who are not pregnant. In fact, endothelial dysfunction as seen in preeclampsia is also seen in vascular disease in non‐pregnant individuals. Also, in non‐pregnant individuals, hypoxia may induce increased endothelial permeability.^25^ These findings suggest that preeclampsia may be consequences of biological responses to hypoxia.

## Shortcomings in our hypothesis

Our hypothesis may be wrong. Feto‐placental hypoxia may not be the underlying cause of preeclampsia in all women. Also, preeclampsia may not develop in all pregnancies with feto‐placental hypoxia. Some women may be genetically more susceptible to preeclampsia than others. Also, paternal genes may influence the risk of preeclampsia and the placental size. It is also likely that metabolic factors and inflammatory factors play roles in the development of preeclampsia. The relationship of metabolic and inflammatory factors in the mother and in the placenta with feto‐placental hypoxia remains insufficiently understood.

The negative association of smoking with preeclampsia cannot easily be explained by our hypothesis. It is conceivable, however, that in women who smoke, there is a strong positive selection to pregnancy success of healthy mothers and embryos with potentials for optimal placental function. Thus, the risk for preeclampsia may be reduced in these selected pregnancies. It is known that women who smoke have lower fertility and higher miscarriage rate than non‐smokers.

## Conclusion

We hypothesise that insufficient oxygen supply from the mother may cause hypoxia in the feto‐placental unit in the last part of pregnancy. In such pregnancies, the placental function is normal, and adequate angiogenic responses may lead to placental enlargement. If the feto‐placental hypoxia is caused by impaired placental development in early pregnancy, the angiogenic responses may not result in placental enlargement and the placenta and the fetus may be growth restricted. We propose that both of these underlying causes of feto‐placental hypoxia could lead to preeclampsia.

## Disclosure of interest

There are no conflicts of interest.
